# "In vitro" and multicolor phenotypic characterization of cell subpopulations identified in fresh human adipose tissue stromal vascular fraction and in the derived mesenchymal stem cells

**DOI:** 10.1186/1479-5876-5-55

**Published:** 2007-10-31

**Authors:** Giuseppe Astori, Francesca Vignati, Silvana Bardelli, Monica Tubio, Mauro Gola, Veronica Albertini, Franco Bambi, Giancarlo Scali, Damiano Castelli, Valeria Rasini, Gianni Soldati, Tiziano Moccetti

**Affiliations:** 1 Cell Therapy Unit, Cardiocentro Ticino, Via Tesserete 48, CH-6900 Lugano, Switzerland; 2 Blood Bank and Cell Therapy Unit, Department of Hematology-Oncology, Children's Hospital A. Meyer, Via L. Giordano 13, 50132-Florence, Italy; 3 Swiss Red Cross Blood Transfusion Service, Via Tesserete 50, CH-6900 Lugano, Switzerland; 4 Laboratory of Cell Biology and Advanced Cancer Therapies, University of Modena and Reggio Emilia, Via del Pozzo, 71, 41100-Modena, Italy

## Abstract

**Background:**

The stromal vascular fraction (SVF) is a heterogeneous cell population derived from the adipose tissue. There is still a lack of information concerning the characterization of the cell subpopulations constituting the SVF as well as its mesenchymal and haematopoietic potential. Furthermore there are great variations in its phenotypical characterization.

**Methods:**

Composition of SVF was investigated by FACS analysis, cytological and "in vitro" assays. We studied CD34+ population by combining FACS with human CFC (colony-forming-cell haematopoietic assay). The endothelial fraction was investigated by quantifying the co-expression of specific markers (CD146, CD105, CD31 and UEA-1). Mesenchymal potential was assessed by CFU-F assay and cultured AT-MSC were characterized by a 5-color FACS analysis. The multipotent differentiation potential (osteogenic, adipogenic and chondrogenic) was investigated both at cellular and molecular level.

**Results:**

We identified in the SVF two CD34+ populations with a marked difference in the intensity of antigen expression, the majority of the cells expressing CD34 at low intensity. Moreover, two CD146+ cell populations were clearly distinguishable in the SVF:a CD146 dim accounting for 9.9% of the total SVF cells and a CD146+ bright cell population accounting for about 39.3%. The frequency of CFC clones was comparable with the one reported for peripheral blood. Endothelial cells account for about 7.7% of the SVF cells. AT-MSC differenced in the osteogenic adipogenic and chondrogenic lineage.

**Conclusion:**

The SVF is not a homogeneous cell population, and its final composition could be influenced both by the flow cytometric technique analysis and the SVF extraction steps. The CFU-F frequency in the SVF was 1/4880, a value about seven times greater than the data reported for bone marrow. The antigenic profile of AT-MSC was comparable with bone-marrow derived MSC. AT-MSC were able to differentiate along the osteogenic adipogenic and chondrogenic lineages. The data here reported, further contribute to the characterization of SVF, a tissue providing an alternative as a source of MSC for clinical applications.

## Background

Adipose tissue is derived from the embryonic mesoderm and consists of a supportive stroma containing a heterogeneous cell population including smooth muscle cells, fibroblast, adipocytes, mast-cells and endothelial cells [1-4]. The stromal vascular fraction (SVF) is a heterogeneous cell population derived from manipulation of adipose tissue including homogenization, enzymatic digestion, differential centrifugation, red blood cells (RBC) lysis and washing. The SVF contains multipotent adipose tissue-derived mesenchymal stem cells (AT-MSC). AT-MSC can be expanded in culture for several passages: the adherent derived cell population maintains its mesenchymal phenotype and its multipotency towards the mesenchymal lineage and can be potentially used in regenerative medicine. Other investigators have evidenced that those cells can be differentiated in vitro in several cell types, such as adipocytes [5], chondrocytes [5,6], osteoblasts [7,8], and cardiomyocytes [9,10].

In most experimental studies, the SVF is extracted by applying the protocol developed by Zuk et al [5,11]. In literature, there is still a lack of information concerning the characterization of the cell subpopulations constituting the SVF, its mesenchymal and haematopoietic potential and, as a consequence, there are great variations in the phenotypical characterization of the crude SVF. It is the case of the percentage of CD34+ positive cells detected in the SVF, that varies among authors from 80% [12] to 3.5% [11]. Moreover, the panel of antigens used for the SVF characterization varied among authors: as a result it is difficult to draw final conclusions on the cell composition of the SVF.

In this study, we investigated the antigen composition of the crude SVF by using a multicolour flow cytometric (FACS) and cytological analysis of the cells. The CD34 antigen expression was investigated on the SVF as well as on CD34+ purified cells by combining several haematopoietic and non haematopoietic markers. As "in vitro" evaluation, the colony-forming cell haematopoietic assay (CFC) was applied to quantify the haematopoietic potential of SVF cells. The composition of the endothelial cell fraction was investigated by quantifying the co-expression of several endothelial markers and the *Ulex europaeus *agglutinin 1 (UEA-1) binding.

Expanded AT-MSC cells at passages two was characterized by applying a 5-color FACS analysis. Their multipotent differentiation potential was confirmed both at cellular and molecular level. Finally, the CFU fibroblast assay was used to evaluate the frequency of mesenchymal progenitors in the SVF fraction.

## Methods

### SVF isolation and expansion

Cells were obtained after informed consent from the resection of subcutaneous fat portions from healthy female donors who underwent breast plastic surgery (n = 6). SVF fraction was separated using a procedure modified from Zuk et al [5,11]. Briefly, the tissue was digested with 0.075% collagenase in phosphate buffered-saline solution (PBS) at pH 7.4 for 45 min at 37°C (Cambrex Bio Science, Walkerville). Mature adipocytes and connective tissues were separated from the cell pellet by centrifugation at 800 ×g, for 10 min at 4°C. The cell pellet was resuspended in erythrocyte lysis buffer (155 mM NH_4_Cl, 10 mM KHCO_3_, 0.1 mM EDTA) and incubated for 10 min at room temperature in the dark. The cell suspension was then filtered through a 100 μm mesh filter (Becton Dickinson) and re-suspended in α-MEM/10%FBS. Nucleated cell count was performed both using trypan blue exclusion test and an automated cell counter (Sysmex K4500, Horger, CH). A portion of the cells was used for the Colony Forming Unit Fibroblast Assay (CFU-F). For the isolation and expansion of AT-MSC, the remaining cells of the SVF were plated in T25 flasks in α-MEM/10% FBS at a density of 100.000 cells/ml. This initial passage of the primary cell culture was referred to as passage 0 (P0). Cells were maintained in media until they achieved 75%–90% confluence. The cells were then replated at a density of 5.000 cells/cm^2 ^in T25 tissue culture flasks. The identity of AT-MSC cells was defined by using the criteria purposed by the ISCT committee [13]: adherence to plastic, specific surface antigen expression and multipotent differentiation potential.

### Colony Forming Unit Fibroblast Assay (CFU-F)

The CFU-F assay was used to evaluate the frequency of mesenchymal progenitors in the SVF [14]. Briefly, cells from the SVF were resuspended in duplicate in 6-wells tissue culture plates at a final concentration of 10.000 cells/cm^2 ^in α-MEM/10%FBS and incubated at 37°C, 5% CO_2_. After 14 days, the cells were washed with May-Grunwald Giemsa. Plates were scored under an inverted microscope (Nikon Eclipse TS100, Japan). Colonies were considered aggregates of more than 50 cells.

### CD34+ cells purification

SVF cells were isolated by using the direct CD34 Progenitor Cell Isolation Kit (Miltenyi Biotec GmbH; Bergisch-Gladbach, Germany) following the manufacturer's protocol. Briefly, the SVF was incubated at 4°C for 30 min with the FcR blocking reagent and with magnetic microbeads coated with anti-CD34 primary antibody (QBEND/10). After washing, CD34+ cells were isolated by slow flow of cell suspension through a separation column placed in a magnetic field. To augment CD34+ cell purity, the separation step was repeated twice.

### Morphological evaluation of the cells

SVF cells (7 × 10^4^/slide) were spotted on cytospin slides by using a cytocentrifuge (Cytospin 3, Shandon Scientific Ltd, England), stained with May-Grunwald Giemsa and observed by using a Nikon Eclipse 50i microscope.

### In vitro differentiation

After primary culture in *control medium *(α-MEM/10% FBS + 50 U/ml Penicillin + 50 μg/ml Streptomycin, BioWhittaker, Walkersville, US), we examined the capacity of the cells to differentiate along osteogenic, adipogenic and chondrogenic lineage. Osteogenic induction: cells at passage 2 were plated at a density of 3.1 × 10^3^/cm^2 ^onto 2-wells chamber slides (BD Biosciences, Franklin Lakes, US). Cells were incubated in the *control medium *for one day to adhere them to the slides and the medium was replaced with Osteogenic Induction Medium from *Osteogenic Differentiation Kit *(Cambrex Bioscience, Ltd, Switzerland). Osteoinductive medium is a basal medium supplemented with dexamethasone, *β*-glycerol phosphate and ascorbic-acid-2-phospahate. On day 21 cells were fixed in 10% formalin for 30' and then osteogenic differentiation was assessed by incubating the fixed cells 10' in Alizarin Red S staining, 2% v/v in distilled water.

For the adipogenic induction, confluent cultures were incubated in *Adipogenic Induction medium *from Adipogenic Differentiation Kit (Cambrex Bioscience), while control cultures where fed with *Adipogenic Maintenance medium *from the same kit. *Induction medium *contains rh-Insulin, Dexamethasone, IBMX and Indomethacin, whereas *maintenance medium *contains only rh-Insulin. Induction was performed by culturing cells 3–4 days in *induction medium*, then 1–2 days in *maintenance medium *for 3 times. Adipogenesis was assessed by Oil O Red staining: cells fixed in 10% formalin were incubated in fresh Oil O Red water solution for 5'.(Fluka, Sigma-Aldrich, Saint Louis, US).

For the chondrogenic differentiation, AT-MSC were gently centrifuged in a 15-ml polypropylene conical tube to form small pellets and cultured 21 days in "differentiation basal medium" (Chondrogenic differentiation kit, Lonza) supplemented with 1 mM sodium pyruvate, 0.17 mM ascorbic acid-2-phosphate, 0.1 μM dexamethasone and 20 μg/ml TGF-*β*3. Every 3–4 days, cells were fed with fresh medium. Chondrogenic pellets were fixed in 10% formalin for 1 h at room temperature. Samples were the embedded in paraffin, sections stained with Alcian Blue and counterstained with Nuclear Fast Red.

### RNA isolation and RT-PCR

Induced and uninduced cell layers were rinsed with D-PBS (BioWhittaker, Walkersville, US) after 21 days of adipogenic or osteogenic induction and immediately lysed using TRIzol (Invitrogen Corp. Carlsbad, CA, US). Total RNA was isolated using RNeasy Isolation Kit (Qiagen AG, Hombrechtikon, DE) and 1 μl was reverse-transcribed using Enhanced AMV Reverse Transcriptase and specific primers as indicated by the manufacturer (Sigma, Saint Louis, US). The resulting cDNA was, then, used as a template for PCR amplification with RedTaq Ready Mix (Sigma); the following genes have been inquired: glyceraldehydes-3 phosphate dehydrogenase (GAPDH) (NM_002046, F: 5'-TTCACCACCATGGAGAAGGC-3', R: 5'-GGCATGGACTGTGGTCATGA-3'), Osteocalcin (OSC), (NM_199173, F: 5'-CATGAGAGCCCTCACA-3', R: 5'-AGAGCGACACCCTAGAC-3'), Osteopontin(OSP), (NM_001040060, F: 5'-TTGCTTTTGCCTCCTAGGCA-3', R: 5'-GTGAAAACTTCGGTTGCTGG-3'), transcription factor PPARγ (NM_005037, F: 5'-TCAGGTTTGGGCGGATGC-3', R: 5'-TCAGCGGGAAGGACTTTATGTATG-3'), lipoprotein lipase (LPL) (NM_000237, F: 5'-GAGATTTCTCTGTATGGCACC-3', R: 5'-CTGCAAATGAGACACTTTCTC-3') and fatty acid binding protein 4 (FABP4) (NM_001442, F: 5'-ATGGGATGGAAAATCAACCA-3', R: 5'-GTGGAAGTGACGCCTTTCAT-3'). All primer sequences were original and determined through established mRNA GeneBank sequences. Glyceraldehydes-3-phosphate dehydrogenase was used as a control for assessing PCR efficiency. Reactions were performed in a T3000 thermal cycler (Biometra, Göttingen, Germany) for 35 cycles. Results were visualised by gel electrophoresis in 1.5% agarose (Promega, Madison, US) in a TAE buffer (Invitrogen) and stained with ethidium bromide (Promega). Reactions were repeated at least twice.

### Flow cytometric analysis of SVF cells

SVF cells were stained in quintuplicate with anti-human monoclonal antibodies against the following antigens: CD31 (clone 5.6E) and CD271 (clone ME20.4-1.H4) (FITC labeled), CD15 (clone VIMC6), CD73 (clone AD2), CD105 (clone 1G2) and CD133 (clone AC133/1) (PE labeled), CD45 (clone J33) (ECD labeled), CD13 (clone Immu103.44), CD14 (clone RMO52), CD146 (clone TEA 1/34), CD90 (clone Thy-1/310), (PC5 labeled) and CD34 (clone 581) (PC7 labeled). For the cell viability test, samples were incubated with 7-AAD. Isotype-matched murine FITC, PE, PC5, PC7 and ECD conjugated immunoglobulin were used as controls. All the antibodies were purchased by Beckman Coulter except for CD15, CD133 (Miltenyi Biotech, Germany) and CD73 (Becton Dickinson). Endothelial cells were further characterized by staining the cells with CD45, CD146 and 10 ug/ml of Ulex lecitin (UEA-1 FITC-labelled, Sigma Aldrich) for 1 h at room temperature in the dark. Huvec cells were used as a positive control.

### Flow cytometric analysis of cultured AT-MSC cells

A 5-color flow cytometric analysis was performed on a Cytomics FC500 cytofluorimeter (Beckman Coulter, Miami, FL) and data were analyzed with CXP software. Cells at passages 2 were analyzed for mesenchymal stem cells markers with the following anti-human monoclonal antibodies: CD31 (clone 5.6E), CD44 (clone J-174), CD29 (clone K20) and CD271 (clone ME20.4-1.H4) (FITC labeled), CD105 (clone 1G2), CD133 (clone AC133/1), CD166 (clone 3A6) and CD73 (PE labeled), CD45 (ECD labeled), CD38 (clone LS198-4-3), CD90 and CD13 (PC5 labeled) and CD34 (PC7 labeled). All Ab were from Beckman Coulter, except CD73 (Becton Dickinson), CD133 and CD271 (Miltenyi Biotec). Isotype-matched antibodies were also used.

### Colony-Forming Cell Haematopoietic Assay

The colony-forming cell haematopoietic assay (CFC) was performed on the crude SVF fraction and on enriched CD34+ cells after immunoselection from the SVF. Cells were cultured in Methocult GF H4434 (Stemcell Technologies Inc., Vancouver, Canada) containing 1% methylcellulose in Iscove's Modified Dulbecco's medium (IMDM), 30% Fetal Bovine Serum, 1% Bovine Serum Albumin, 10 × 10^-4 ^M 2-Mercaptoethanol, 2 mM L-glutamine, 3 U/ml recombinant human erythropoietin, 50 ng/ml rh Stem Cell Factor, 10 ng/ml rh GM-CSF and 10 ng/ml rh IL-3. Briefly, the SVF crude cells were plated in duplicate at a final concentration of 100.000 cells/ml and the SVF-CD34+ selected cells were plated at a final concentration of 10.000 cells/ml in tissue culture plates (Costar, Acton, MA). Plates were incubated at 37°C in a fully humidified atmosphere of 5% carbon dioxide and, after 14 days, each culture plate was examined under an inverted microscope. Granulocyte-erithrocyte-macrophage-megakariocyte colony-forming units (CFU-GEMM), granulocyte-macrophage colony-forming units (CFU-GM) and blast-forming units erythroid/mix (BFU-E/mix) were identified and counted using standard criteria. Colonies were considered aggregates of more than 50 cells.

## Results

### Isolation and characterization of SVF and AT-MSC cells

The SVF consists of a heterogeneous cell population: based on the antigen expression, it is possible to individuate a subset of blood-derived cells. CD45 was detected with great variability on a mean percentage of 9.0 ± 6.9% of the SVF cells (range 1.5–17.4). The CD14, expressed on the monocytic lineage, was detected on 10.9 ± 9.6% of the cells (range 4.1–17.7) and CD13, expressed both on the early committed progenitors and mature granulocytes and monocytes was detected in 5.6 ± 3.9% of the cells (range 2.8–8.4). CD90, a marker associated with haematopoietic stem cells but also on connective tissue, was expressed on 29.2 ± 20.8% of the SVF (range 10.3–49.9). Complete data are reported in Table 1 and Table 2.

**Table 1:** percentage of expression of surface antigens on the cells constituting the crude SVF (n = 6)

***ANTIGENS***	***CD13***	***CD14***	***CD15***	***CD31***	***CD34***	***CD45***	***CD73***	***CD90***	***CD105***	***CD133***	***CD146***	***CD 271***
MEAN %	5.6	10.9	2.0	1.8	6.9	9.0	1.6	29.2	24.1	1.2	47.1	0.6
**SD**	3.9	9.6	1.7	1.5	3.0	6.9	1.5	20.8	22.1	1.5	3.2	0.5

**Table 2:** percentage of co-expression of surface antigens on the cells constituting the crude SVF (n = 6)

***ANTIGENS***	***CD45/34***	***CD45/133***	***CD34/90***	***CD34/133***	***CD105/146***	***CD31/146***	***CD31/105***	***CD31/15***	***CD31/90***	***CD90/73***
MEAN %	2.0	0.4	7.0	6.8	6.7	1.9	2.4	1.2	2	2.1
**SD**	2.2	0.1	5.9	0.5	1.2	0.3	0.6	1.1	0.7	0.3

**Figure 1:**
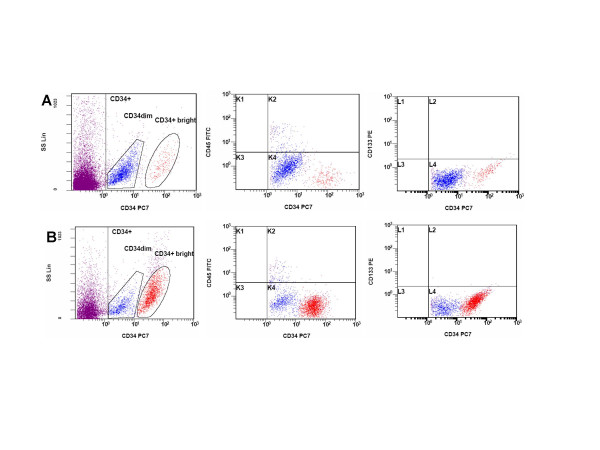
FACS analysis of haematopoietic and endothelial cell subsets in the SVF. (A) CD34+ cells were analyzed in co-expression with CD45 and CD133 cell markers on crude SVF cell population (n = 6) and (B) after CD34 cell selection (n = 3).

The percentage of SVF cells expressing CD34 has been reported with great variability among authors [11,12,15,16]. In the present study, 6.9 ± 3.0% (range 3.4–10.2) of the SVF cells expressed the CD34 antigen. Haematopoietic progenitor cells are defined by the co-expression of the CD45+ cell marker: interestingly, when analyzing the co-expression of this antigen on CD34+ cells, only 2.0 ± 2.2% (range 0.4–3.5) of the CD34+ cells stained for CD45. 7.0 ± 5.9% (range 2.8–11) of the CD34+ cells expressed the CD90, a cell marker present on haematopoietic stem cells [17]. The CD133 cell marker is co-expressed on a variable percentage of CD34+ haematopoietic progenitor cells, on stem cells [18], on developing epithelium and on the haemangioblast [19]. In our cell populations, CD133 was expressed in only a small percentage of cells on the SVF (1.2 ± 1.5, range 0.40–3.80). To better characterize the antigenic profile of CD34+ cell subpopulation, we purified the CD34 cells by immunomagnetic selection. Cells were then subjected to FACS analysis and CFU haematopoietic assay. Two CD34+ cell subpopulations were present in the SVF fraction: a CD34 dim, accounting for about 90% of the CD34+ cells, and a CD34+ bright cell subpopulation (Figure 1). Among them, the CD45+ cells are mainly represented in the CD34 dim subpopulation (11.3%). The CD133+ is coexpressed by only 0.3% of the CD34+ cells. It is possible to conclude that only a small percentage of the SVF cells expressing the CD34+ antigen are haematopoietic progenitors.

**Figure 2:**
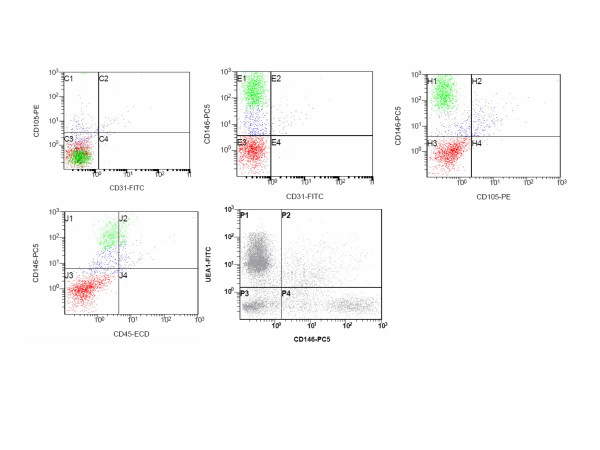
Endothelial cell markers expression on primary SVF cell population. The cells represented in the CD146 vs UEA-1 plot are CD45-.

Endothelial cell-associated markers, including CD31 [20], CD105 [21] and CD146 are expressed on SVF cells. The CD146 [22], a cell marker expressed on mature endothelia, pericytes, endothelial progenitors cells (EPC) and at low intensity on a subset of activated T lymphocytes [23] stains 47.1 ± 3.2 of the cells (range 44.8–49.3) As can be observed in Figure 2, two distinct CD146 dim and CD146 bright cell clusters can be clearly distinguished. To finally confirm that mature endothelial cells are represented in the SVF, we have stained the cells in triplicate with CD45, CD146 and UEA-1. The results obtained suggest that it is possible to identify in the SVF a cell population having an endothelial phenotype defined as CD45-, CD146+ and UEA-1+ accounting for about the 7.7% of the total cells. (Figure 2).

**Figure 3:**
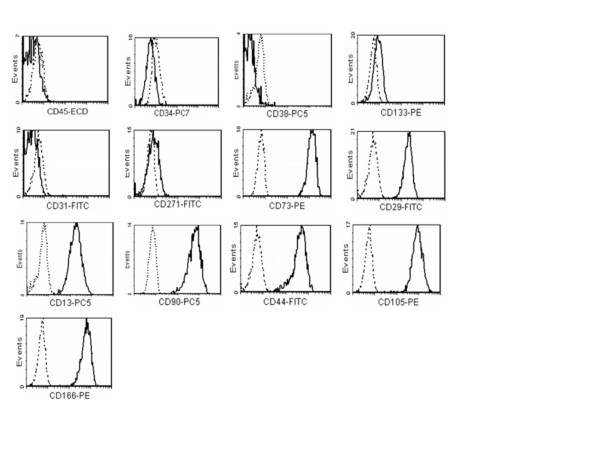
expression of surface markers in AT-MSC cells derived from SVF cells expansion (n = 5).

Adherent cells generated after SVF expansion were observed after 4–7 days in culture. Cells rapidly generate a monolayer of fibroblastic-like morphology. At the second passage cells were analyzed and resulted to be uniformly negative (≤ 2%) for haematopoietic CD34, CD38, CD45, CD133, and non haematopoietic markers CD31 and CD271, and to be positive (≥ 95%) for CD13, CD29, CD44, CD73, CD90, CD105 and CD166 (Figure 3).

### In vitro differentiation and molecular analysis

We evaluated both osteogenic, adipogenic and chondrogenic potential in AT-MSC at the second passage. For the osteogenic differentiation, morphological changes appeared during the second week of subculture. At the end of the 21-day induction period, some calcium crystals were clearly visible in the culture. Cell differentiation was confirmed by Alizarin Red staining. Osteogenic cell induction was confirmed at the molecular level: induced cells showed an upregulation of the expression of osteocalcin and osteopontin genes with RT-PCR assay.

**Figure 4:**
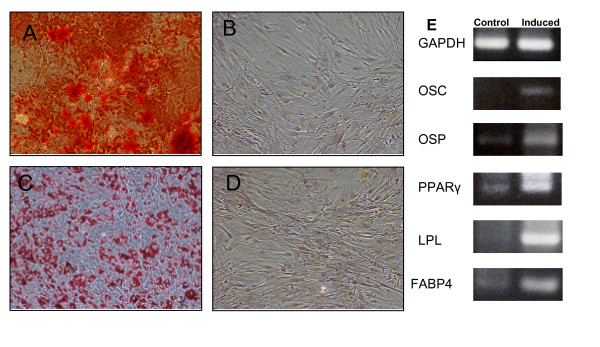
osteogenic and adipogenic differentiation of adherent cells from SVF culture. A: osteogenic differentiation (Alizarin Red staining); B: control; C: adipogenic differentiation (Oil O Red staining); D: control (100×). E: gene expression profile of un-induced cells compared with cultured ASC following osteogenic and adipogenic induction. GAPDH: glyceraldehide phosphate dehydrogenase; OSC: osteocalcin; OSP: osteopontin; PPARγ: peroxisome proliferator-activated receptor γ; LPL: lipoprotein lipase; FABP4: fatty acid binding protein 4.

The adipogenic potential was assessed by induction of confluent AT-MSC at second passage. At the end of the induction cycles, a consistent cell vacuolation was evident in the induced cells. Vacuoles brightly stained for fatty acid with Oil O Red staining. At the molecular level, induced cells showed differential expression of genes from the adipogenic pathway (LPL, FABP4 and PPARγ) (Figure 4).

**Figure 5:**
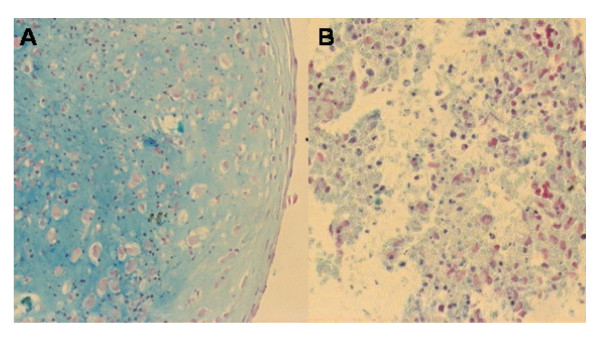
chondrogenic differentiation of cultured AT-MSC by micromass method. A: induced cells; B: control; Alcian Blue staining (20×).

Chondrogenic potential was assessed by induction of AT-MSC using the micromass method. Control cells did not even retain a spheroid shape and showed no specific staining while induced cells had a strong signal from Alcian Blue (Figure 5)

### Morphology

**Figure 6:**
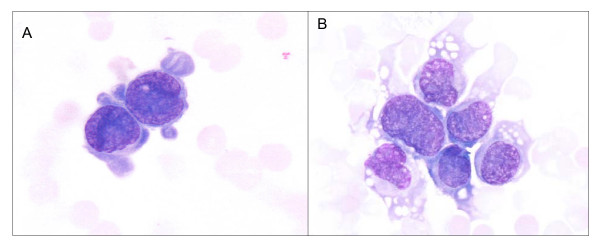
SVF cells spotted on a cytospin slide. It is possible to observe the presence of immature cells (A) together with cells having a more differenciated morphology (B). May-Grunwald Giemsa staining (100×).

Morphologic analysis of the SVF revealed the presence of immature cells together with cells having a more differenciated morphology (Figure 6). May-Grunwald Giemsa staining (Figure 6, 100×).

### Colony Forming Unit Fibroblast and CFC assay

The CFU-F assay was used to evaluate the frequency of mesenchymal progenitors in the SVF fraction. The estimated frequency of AT-MSC in the SVF was 1 in 4880 ± 1650 SVF cells (range 1826–6774).

**Figure 7:**
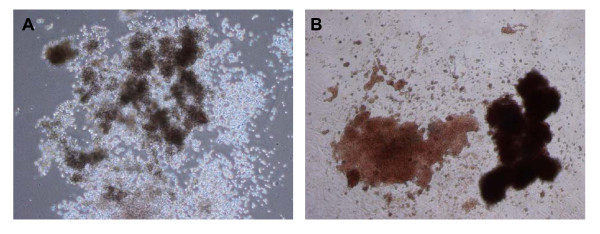
phase-contrast photomicrographs of haematopoietic clones in Methocult obtained from the SVF. A: large CFU-GM (50×); B: large BFU-E (50×).

To assess the haematopoietic potential of the SFV, the crude fraction (n = 5) and the CD34+ selected cells (n = 3) were plated in Methocult GF H4434. The frequency of haematopoietic colonies was comparable in the two cell fractions. Overall, the colonies were isolated with the following frequency (per 10^5 ^cells): CFU-E, 3.3 ± 8.2 (range 0–20); BFU-E, 3.2 ± 6.0 (range 1–15); CFU-GM, 3.6 ± 8.1 (range 1–20); CFU-GEMM, 5.0 ± 12.3 (0–30). Images of haematopoietic colonies isolated from SVF are reported in Figure 7.

## Discussion

The aim of this study was the analysis of the SVF and the derived AT-MSC population by using a cytological, flow-cytometrical (FACS) and *in vitro *assay. We speculate some hypothesis on the reason why, among authors, there are marked variations in the percentage of detection of different cell antigens by FACS: since the SVF is not a homogeneous cell population, its final composition could be influenced both by the FACS technique analysis and the cellular extraction steps. For the latter, it is likely that the cells constituting the SVF have different buoyant densities: mature cells (i.e. mature epithelia) are characterized by an increased cell density if compared with immature cells. As a consequence, the centrifugal force, the centrifugation time and temperature and the resuspension media, could influence its final composition.

We found a great variation in the percentage of haematopoietic CD45 cells in the SVF: since each fat cell in white adipose tissue is in contact with at least one capillary, providing a vascular network that allows continued growth of the tissue [24], the variations in CD45 expression could reflect differences in the vascularization of the starting tissue. Furthermore it is always possible to observe in the starting material a different level of contamination with peripheral blood depending from the tissue harvesting procedure. Moreover, it has been reported that also the yield and growth characteristics of adipose-derived mesenchymal stem cells are affected by the tissue-harvesting procedure [25].

The variations in the percentage of detection for certain cell antigens of more than 22 times among authors (it is the case of CD34+ cells) could be explained in the different gating strategies applied during the flow cytometric characterization. The CD34, a heavily glycosylated type I transmembrane protein, is expressed on early lymphohaematopoietic stem and progenitor cells: since the SVF is constituted by a portion of haematopoietic as well as endothelial-derived cells, the CD34+ antigen should be investigated by the simultaneous expression of other lineage-associated molecules (i.e. CD45) in order to determine the origin of CD34+ cells. We identified two CD34+ cell populations characterized by a striking difference in the intensity of antigen expression, the great majority of the cells expressing the CD34 at low intensity. Interestingly, the CD133 is co-expressed by CD34+ cells in only the 6.8% of the cases.

To better investigate the haematopoietic origin of CD34+ cells in the SVF, we enumerate the haematopoietic progenitors by using a CFU in vitro assay. All the types of cell progenitors were isolated from the SVF: the frequency of isolation was generally low. If the number of haematopoietic clones is reported to the percentage of CD45+ cells detected in the SVF, one can estimate the frequency of CFU related to the haematopoietic cells in the SVF (× 100.000): CFU-E: 36.8; BFU/E: 35.7; CFU-GM: 40.2; CFU-GEMM: 55.8. It is possible to conclude that the frequency of haematopoietic clones isolated in the SVF, could be compared with the data reported for the normal peripheral blood [26]. Based on those observations, it is likely that they could be derived from the peripheral blood cells circulating in the adipose tissue.

Aiming to investigate the composition of the endothelial markers in the SVF, we have stained the cells in triplicate with CD45/CD146/UEA-1. The results obtained suggests that endothelial cells are represented in the SVF accounting for about 7.7% of SVF cells.

It is likely that a percentage of endothelial progenitor cells are also present in the SVF: *in vitro*, we observed the formation of cell clones possessing a specific endothelial-like morphology in the CFU assay, but further specific analysis must be done (data not shown).

The CD271 LNGFR (low-affinity nerve growth factor receptor), has been detected on bone marrow stromal cells, [27,28] follicular dendritic cells [29] and on mesenchymal cells involved in mesenchymal-epithelial interactions [30], and it has been recently purposed as a cell marker for MSC isolation [28]. The presence of CD271 in the SVF has not been investigated yet: in our experience, CD271 was detected on 0.6 ± 0.5% of the SVF cells (range 0.2–1.2). We also investigated the expression of CD271 on expanded AT-MSC at second passage: we conclude that there is no significant variation in the expression of this cell antigen in AT-MSC expanded cells compared to crude SVF.

The AT-MSC could be used for cell therapy protocols: many groups have evidenced that those cells can be differentiated in vitro in several cell types; among them adipocytes [5], chondrocytes [5,6], osteoblasts [7,8], and cardiomyocytes [9,10]. AT-MSC possess a mesenchymal phenotype confirmed in our study by applying the minimal criteria for defining multipotent mesenchymal stromal cells as stated by the International Society for Cellular Therapy [13] both at cellular and molecular level. The CFU-F assay was used to evaluate the frequency of mesenchymal progenitors in the SVF estimating a frequency of 1/4880, a value about seven times greater than the reported data for bone marrow [31,32] and comparable with the data reported for umbilical cord blood [31,33].

The SVF already find application in cellular-based protocols for reconstructive surgery [34] and cardiovascular disease treatment : the data here reported, further contribute to the characterization of this cell population, now constituting an alternative as a source of MSC for clinical applications.

## Competing interests

The author(s) declare that they have no competing interests.

## Authors' contributions

GA designed the study and drafted the manuscript. FV carried out the cell differentiation experiment, molecular genetics and and FACS analysis. SB carried out cell extraction and expansion. MT participates in the molecular genetic studies. VA participates in the FACS analysis. GS and DC authorized access to blood donor samples and carried out some tissue staining; VR carried out some of the tissue staining. GSO, MG, FB and TM contributed clinical expertise and oversight to the study design. All authors read and approved the final manuscript.
